# Disease and social factors associated with healthcare utilization for the treatment of SARS-CoV-2 infections in a longitudinal cohort of essential workers in Arizona

**DOI:** 10.1186/s12913-023-10064-y

**Published:** 2023-10-18

**Authors:** Patrick Rivers, Krystal Jovel, Ferris Ramadan, Jared Joshua Anucha Barnett, Katherine D. Ellingson, Jefferey L. Burgess, Karen Lutrick

**Affiliations:** 1 College of Medicine, University of Arizona, Tucson, USA; 2 Mel and Enid Zuckerman College of Public Health, University of Arizona, Tucson, USA; 3 Department of Family and Community Medicine, University of Arizona, 655 N. Alvernon Way, Tucson, AZ 85712 USA

**Keywords:** COVID-19, Healthcare utilization, Public health

## Abstract

**Background:**

Demands on health systems due to COVID-19 are substantial, but drivers of healthcare utilization are not well defined in non-severe SARS-CoV-2 infections. Among a prospective cohort of frontline workers from July 2020 to February 2023, we assessed predictors of healthcare utilization during SARS-CoV-2 infection.

**Methods:**

Weekly specimens tested via real-time reverse transcriptase polymerase chain reaction analysis. Participants reported sociodemographic, health status information, and illness experience information. Primary outcome was healthcare utilization during SARS-CoV-2 infection. Predictors included sociodemographic characteristics, baseline health status, and measures of illness severity. Multivariable logistic regression was utilized to generate odds ratios for predictors of healthcare utilization.

**Results:**

1,923 SARS-CoV-2 infections (1,276 first infections and 647 reinfections from 4,208 participants): 1221 (63.5%) individuals were between 40 and 65 years old; 1115 (58.0%) were female; 449 (23.3%) were Hispanic and 1305 (67.9%) non-Hispanic White. 294 (15.3%) individuals sought medical care during first infection, 106 (5.5%) during reinfection. Sociodemographic and baseline health characteristics were not associated with healthcare utilization during infections from any variant for first infections, while age (OR 1.04, 95%CI 1.01–1.07) was during Omicron reinfection. In first infection, number of symptoms (OR 1.16, 95%CI 1.00-1.36 in Origin/Alpha, OR 1.12, 95%CI 1.00-1.49 in Delta, OR 1.09, 95%CI 1.01–1.16 in Omicron), number of days spent in bed (OR 1.13, 95%CI 1.02–1.33 in Origin/Alpha, OR 1.23, 95%CI 1.00-1.59 in Delta, OR 1.12, 95%CI 1.03–1.22 in Omicron), and illness duration (OR 1.01, 95%CI 1.00-1.04 in Origin/Alpha, OR 1.01, 95%CI 1.00-1.03 in Delta, OR 1.01, 95%CI 1.00-1.02 in Omicron) were related to healthcare utilization for all variants. Number of days in bed (OR 1.12, 95%CI 1.01–1.27), illness duration (OR 1.01, 95%CI 1.00-1.02), and hours of work missed (OR 2.24, 95%CI 1.11–4.74) were positively associated with healthcare utilization during Omicron reinfection.

**Conclusion:**

The main factors associated with healthcare utilization for SARS-CoV-2 infection were symptom severity and duration. Practices and therapeutics aimed at decreasing these factors would be most helpful in easing the burden on health systems.

## Background

The COVID-19 pandemic has placed an unprecedented demand on the United States’ (US) health care system and continues to be substantial burden nationally. Since August 2020, 5.9 million people in the US were admitted to hospitals for COVID-19 [1]. Further, nearly half of US adults have either delayed or avoided accessing routine medical care [2]. Utilization of the healthcare system by individuals with non-severe COVID-19 illness has not been well documented. In order to adequately prepare for future pandemics and the ongoing COVID-19 epidemic, it is important to understand drivers of healthcare utilization during mild-to-moderate respiratory illnesses like COVID-19. While the risk of death from the Omicron (B.1.1.529) variant is lower than previous variants, the overall number of cases, emergency department visits, and hospital admissions is higher [3, 4]. Healthcare utilization and COVID-19 outcomes are not uniform, and a better understanding of patients who may require additional care following SARS-CoV-2 infection is important [5].

Differences by age, race, ethnicity, gender, underlying medical conditions, and insurance status have been found in rates of delaying or avoidance of medical care, with differences also manifesting in overall healthcare utilization for both hospitalized and non-hospitalized patients once medical care is sought [6–8]. Non-White individuals, men, and uninsured individuals were more likely to delay or avoid care; and older individuals, women, those with higher BMI, former smokers, and individuals with a greater number of pre-existing conditions were all associated with higher healthcare utilization. As health systems have struggled to provide care during the pandemic, quality of care for African American and Hispanic patients has dropped, [9–11] while their rate of hospitalization has increased [12]. A better understanding of differences between sociodemographic characteristics and illness-related factors that influence healthcare utilization can help inform resource allocation and ongoing health service needs – of particular importance during surges in COVID-19 and periods of high healthcare utilization.

The Arizona Healthcare Emergency Response and Other Essential Workers Surveillance (AZ HEROES) Study is a longitudinal cohort study of 4,000 essential workers in Arizona [13]. The primary objective of this paper is to examine healthcare utilization for essential workers who experienced SARS-CoV-2 infection during the study period. We examined predictors of healthcare utilization and differences by sociodemographic characteristics, health status, and illness experience.

## Methods

### Study design and population

The AZ HEROES study began in July 2020 as a longitudinal, prospective cohort funded by the Centers for Disease Control and Prevention (CDC), recruiting over 4200 essential workers in Arizona [13]. Data collected through February 8, 2023 were included in this analysis. Enrollment groups included healthcare personnel, first responders, and other essential workers across the state of Arizona who worked a minimum of 20 hours per week and had regular close-contact exposure to coworkers or the public. All study participants provided written informed consent, and the study protocol was reviewed and approved by the University of Arizona IRB (reference number 2,006,729,444) and the CDC.

### Data collection

Upon enrollment, participants completed a survey in which they self-reported sociodemographic information (including gender, age, race/ethnicity, education, household income, and occupation), health information (including medical conditions, height and weight, tobacco use, and daily medications use), and SARS-CoV-2 infection history. Each week, participants completed text message surveys and reported onset of COVID-like symptoms – including fever, chills, cough, shortness of breath, sore throat, diarrhea, muscle aches, or a change in smell or taste [13, 14].

Participants submitted self-collected mid-turbinate nasal swabs each week of study participation. If participants experienced an onset of illness with COVID-like symptoms outside their regular swab collection, they completed an additional illness swab. Both weekly and illness swabs were tested by Marshfield Clinical Research Laboratories (Marshfield, WI, USA) via qualitative reverse-transcriptase-polymerase-chain-reaction (RT-PCR) assays to detect the presence of SARS-CoV-2. Whole-genome sequencing was completed on specimens positive for SARS-CoV-2 with a cycle threshold value < 30 (the number of cycles required for the fluorescent signal to cross the threshold or exceed background level) to determine variant. Specimens positive for SARS-CoV-2 but ineligible for sequencing – those with a cycle threshold ≥ 30 – variant predominance was assigned using the date of infection and which variant accounted for more than 50% of infections according to CDC data [15].

Participants who reported symptoms during weekly surveillance, submitted an illness swab, or submitted any sample that was positive for SARS-CoV-2, were prompted to complete an acute illness survey, an illness update survey (if the illness lasts longer than one week), and a final illness survey when symptoms resolved [13, 14] The illness surveys gather information on illness symptoms, severity (e.g. number of hours of work missed and number of days spent at least half in bed), medical utilization, and illness duration.

Beginning in December 2020, participants were prompted to report uptake of the COVID-19 vaccine. Participants received a survey at least every eight weeks until reporting a completed two-dose series. Beginning in September 2021, participants who had completed their initial series at least eight weeks prior began receiving a survey to report COVID-19 boosters. Participants received the survey at least every eight weeks throughout the duration of the study period to be able to report additional booster shots, with participants completing their initial series during this time also asked about boosters. Study staff confirmed vaccination doses and dates in post-infection calls to participants. Vaccination was verified by participant-provided vaccination cards and the Arizona State Immunization Information System, as available. Vaccination status at infection was derived by comparing their date of infection to the dates of all vaccinations, if available.

### Analysis

Participants who did not test positive for SARS-CoV-2 during the study period were excluded from analysis. The primary outcome was healthcare utilization during the course of a SARS-CoV-2 infection. Healthcare utilization was defined as accessing medical care from an emergency department, hospital, outpatient clinic, telehealth, or other medical care setting – as self-reported by participants. Healthcare utilization was derived using answers to all surveys completed by the participant during the course of their illness. The SARS-CoV-2 infection was defined as the period between onset of symptoms or positive SARS-CoV-2 test result (whichever came first) until the cessation of symptoms and a self-reported health of 90% normal health or better. First SARS-CoV-2 infection was defined as those from participants who entered the study reporting no prior SARS-CoV-2 infection, and the first in-study positive. Reinfection SARS-CoV-2 infection was all in-study positive infections for a participant who entered the study having had a prior SARS-CoV-2 infection, or all in-study positive infections after the first for participants entering the study having no prior SARS-CoV-2 infection. Infections were deemed “reinfections” if at least 90 days had elapsed since the date of last positivity for any variant, or at least 45 days if the infections were two different variants.

Independent variables included sociodemographic characteristics (age, gender, race/ethnicity, and education), occupation category, vaccination status at time of infection, SARS-CoV-2 variant of infection (presumed or confirmed), health characteristics (number of chronic conditions, body mass index (BMI), smoking status, and number of daily medications), and illness characteristics (number of symptoms, work missed, days spent in bed, and illness duration). These variables were chosen a priori based on previous literature [6–8] or their believed theoretical association with healthcare utilization.

### Statistical analysis

Independent variables listed previously were stratified by healthcare utilization and type of SARS-CoV-2 infection (first or reinfection). Pearson’s chi-squared tests and Fisher’s exact tests were used to determine unadjusted differences between hospital utilization groups with statistical significance based on p-values < 0.025 to account for a Bonferroni correction for multiple comparisons. For the main analysis, multivariable logistic regression models with robust standard errors [16] were used to identify factors associated with healthcare utilization. Multivariable models were stratified by variant to account for differences in the timing of variant predominance and adjusted for all independent variables of interest. All assumptions of the logistic regression models were checked, and none were found to be violated. Statistical significance was based on 95% confidence intervals (95% CI). All statistical analyses were completed using R, version 4.1.2 (2021, R Foundation for Statistical Computing, Vienna, Austria).

## Results

### Participant characteristics

Between July 2020 and February 2023, a total of 1,923 SARS-CoV-2 infections occurred in 4,208 AZ HEROES participants; 1,276 (66%) were first infections and 647 (34%) were reinfections (Table 1). Over 74% (n = 945) of first infections were from the Omicron variant. At the time of infection in nearly half (n = 612, 48%) of these infections, the participant was booster-vaccinated, approximately one quarter were unvaccinated or vaccinated with their initial series only (n = 301, 24% and n = 320, 25% respectively), and only 3% (n = 43) were partially vaccinated (received only one dose of a non-Johnson & Johnson vaccine). 62% (n = 801) were 40–65 years of age, 58% (n = 745) were female, 69% (n = 877) were non-Hispanic White, and 22% (n = 285) were Hispanic. Participants were relatively evenly split between healthcare personnel (n = 481, 38%), first responder (n = 324, 25%), and other essential worker (n = 471, 37%). The majority of the cohort did not report presence of any chronic conditions (n = 837, 66%) and approximately half did not report taking any daily medications (n = 629, 49%).

**Table 1:** Characteristics of 1923 SARS-CoV-2 infection during enrollment in AZ HEROES, July 2020 to February 2023

			First positive (n = 1276, 66.4%)					Reinfection (n = 647, 33.6%)				
Characteristic	Total (N = 1923)		Did not seek medical care		Did seek medical care			Did not seek medical care		Did seek medical care		
			(n = 982, 51.1%)		(n = 294, 15.3%)			(n = 541, 28.1%)		(n = 106, 5.5%)		
	**No. or Mean**	**Col % or SD** ^**a**^	**No. or Mean**	**Row % or SD**	**No. or Mean**	**Row % or SD**	**p-value** ^**b**^	**No. or Mean**	**Row % or SD**	**No. or Mean**	**Row % or SD**	**p-value** ^**b**^
**Variant of infection**							< 0.001					0.025
Origin/Alpha^c^	236	12.3%	151	64.0%	59	25.0%		17	7.2%	9	3.8%	
Delta	143	7.4%	76	53.1%	45	31.5%		17	11.9%	5	3.5%	
Omicron	1544	80.3%	755	48.9%	190	12.3%		507	32.8%	92	6.0%	
**Vaccination status at infection**							0.125					0.097
Unvaccinated	500	26.0%	219	43.8%	82	16.4%		174	34.8%	25	5.0%	
Partially vaccinated with initial series	55	2.9%	31	56.4%	12	21.8%		8	14.5%	4	7.3%	
Completed initial series	510	26.5%	257	50.4%	63	12.4%		153	30.0%	37	7.3%	
Booster vaccinated	858	44.6%	475	55.4%	137	16.0%		206	24.0%	40	4.7%	
**Age**							0.290					0.008
18–39 years	628	32.7%	345	54.9%	89	14.2%		175	27.9%	19	3.0%	
40–59 years	1080	56.2%	540	50.0%	172	15.9%		300	27.8%	68	6.3%	
60 + years	215	11.2%	97	45.1%	33	15.3%		66	30.7%	19	8.8%	
**Gender**							0.002					< 0.001
Female	1115	58.0%	549	49.2%	196	17.6%		291	26.1%	79	7.1%	
Male	799	41.5%	429	53.7%	96	12.0%		247	30.9%	27	3.4%	
Missing	9	0.5%	4	44.4%	2	22.2%		3	33.3%	0	0.0%	
**Race/Ethnicity**							0.276					0.449
NH White^d^	1305	67.9%	686	52.6%	191	14.6%		360	27.6%	68	5.2%	
NH Black	60	3.1%	36	60.0%	7	11.7%		16	26.7%	1	1.7%	
NH Asian	41	2.1%	21	51.2%	9	22.0%		8	19.5%	3	7.3%	
Hispanic	449	23.3%	209	46.5%	76	16.9%		132	29.4%	32	7.1%	
Other	42	2.2%	18	42.9%	8	19.0%		15	35.7%	1	2.4%	
Missing	25	1.3%	12	48.0%	2	8.0%		10	40.0%	1	4.0%	
**Education**							0.876					0.131
Elementary or high school, no diploma	1	0.1%	1	100.0%	0	0.0%		0	0.0%	0	0.0%	
High school diploma or GED	54	2.8%	28	51.9%	7	13.0%		16	29.6%	3	5.6%	
Some college	345	17.9%	158	45.8%	49	14.2%		124	35.9%	14	4.1%	
College degree or above	1441	74.9%	761	52.8%	225	15.6%		371	25.7%	84	5.8%	
Missing	82	4.3%	34	41.5%	13	15.9%		30	36.6%	5	6.1%	
**Occupation category**							0.675					0.004
Health Care Personnel^e^	702	36.5%	366	52.1%	115	16.4%		185	26.4%	36	5.1%	
First Responder^f^	503	26.2%	255	50.7%	69	13.7%		162	32.2%	17	3.4%	
Other Frontline/ Essential Worker^g^	718	37.3%	361	50.3%	110	15.3%		194	27.0%	53	7.4%	
**# of chronic conditions**							< 0.001					0.060
0	1237	64.3%	679	54.9%	158	12.8%		346	28.0%	54	4.4%	
1	454	23.6%	209	46.0%	81	17.8%		131	28.9%	33	7.3%	
2	120	6.2%	50	41.7%	30	25.0%		31	25.8%	9	7.5%	
3	35	1.8%	15	42.9%	10	28.6%		6	17.1%	4	11.4%	
4+	10	0.5%	3	30.0%	3	30.0%		3	30.0%	1	10.0%	
Missing	67	3.5%	26	38.8%	12	17.9%		24	35.8%	5	7.5%	
**Body mass index**							0.037					0.068
Less than 18.5	8	0.4%	4	50.0%	1	12.5%		3	37.5%	0	0.0%	
18.5–24.9	611	31.8%	341	55.8%	85	13.9%		155	25.4%	30	4.9%	
25–29.9	695	36.1%	367	52.8%	100	14.4%		202	29.1%	26	3.7%	
30–39.9	450	23.4%	206	45.8%	78	17.3%		128	28.4%	38	8.4%	
Greater than 40	68	3.5%	27	39.7%	16	23.5%		20	29.4%	5	7.4%	
Missing	91	4.7%	37	40.7%	14	15.4%		33	36.3%	7	7.7%	
**Smoking status (tobacco and vaping)**							0.089					0.754
Never Smoked	1461	76.0%	760	52.0%	210	14.4%		408	27.9%	83	5.7%	
Former or Current Smoker	395	20.5%	196	49.6%	72	18.2%		109	27.6%	18	4.6%	
Missing	67	3.5%	26	38.8%	12	17.9%		24	35.8%	5	7.5%	
**# of daily medications**							< 0.001					0.007
0	921	47.9%	523	56.8%	106	11.5%		259	28.1%	33	3.6%	
1	381	19.8%	200	52.5%	54	14.2%		101	26.5%	26	6.8%	
2	246	12.8%	104	42.3%	52	21.1%		73	29.7%	17	6.9%	
3	128	6.7%	53	41.4%	32	25.0%		38	29.7%	5	3.9%	
4	86	4.5%	37	43.0%	15	17.4%		23	26.7%	11	12.8%	
5+	87	4.5%	32	36.8%	23	26.4%		23	26.4%	9	10.3%	
Missing	74	3.8%	33	44.6%	12	16.2%		24	32.4%	5	6.8%	
**# of illness symptoms**							< 0.001					< 0.001
0	250	13.0%	118	47.2%	15	6.0%		110	44.0%	7	2.8%	
1–5	493	25.6%	270	54.8%	34	6.9%		169	34.3%	20	4.1%	
6–10	852	44.3%	448	52.6%	141	16.5%		207	24.3%	56	6.6%	
10+	308	16.0%	136	44.2%	103	33.4%		46	14.9%	23	7.5%	
Missing	18	0.9%	8	44.4%	1	5.6%		9	50.0%	0	0.0%	
**# of hours of work missed**	43.1	33.6	40.8	26.4	57.3	49.7	< 0.001	32.4	19.4	42.5	30.4	0.013
**# of days spent at least half in bed**	2.4	3.0	2.1	2.4	4.1	4.1	< 0.001	1.6	2.3	3.3	3.7	< 0.001
**Illness duration (# of days)**	22.1	23.8	22.2	22.3	27.3	27.7	0.004	17.4	21.6	30.1	31.8	< 0.001

a. SD = Standard deviation

b. p-values based on Pearson’s chi-squared tests for variables in which all cells have a frequency of 5 or greater, and Fisher’s exact test for variables with cells of less than 5 individuals. P-values 0.025 or less are considered statistically significant due to a Bonferroni correction for multiple comparisons.

c. Origin and Alpha combined due to very few infections during Alpha variant predominance

d. NH = Non-Hispanic

e. Includes individuals working in inpatient, outpatient, or long-term care healthcare facilities

f. Includes firefighters, emergency medical services workers, law enforcement, border patrol, and correctional officers

g. Includes individuals working in education, retail, food service, hospitality, infrastructure, manufacturing, utility, and transportation

Over 90% (n = 599) of reinfections that occurred during the study period were from Omicron variants. At the time of reinfection, 38% (n = 612) of the participants were booster-vaccinated, 31% (n = 199) were unvaccinated, 25% (n = 160) were vaccinated with their initial series only, and 2% (n = 12) were partially vaccinated. At the time participants had a SARS-CoV-2 reinfection, 65% (n = 420) were 40–65 years of age, 57% (n = 370) were female, 66% (n = 428) were non-Hispanic White, and 25% (n = 164) were Hispanic. 34% were healthcare personnel (n = 221), 28% first responder (n = 179), and 38% other essential worker (n = 247) occupations. 62% had no chronic conditions (n = 400), with nearly half taking no daily medications (n = 292, 45%).

Across all SARS-CoV-2 infections, the average illness duration was 22.1 days (SD 23.8), and participants missed an average of 43.1 hours of work (SD 33.6) and spent 2.4 days in bed (SD 3.0), with nearly half (n = 852, 44%) experiencing 6–10 illness symptoms.

### Factors associated with healthcare utilization

Of all participants experiencing a first SARS-CoV-2 infection during the study, 294 (23%) reported seeking medical care and 982 (77%) did not (Table 1). In unadjusted analyses, participants who sought medical care for their first SARS-CoV-2 infection were (statistically) significantly more likely to be infected by the Delta (B.1.617.2) variant or Origin/Alpha (B.1.1.7) than Omicron (p < 0.001) and were more likely to be female (p = 0.002). They had higher numbers of chronic conditions (p < 0.001) and took more daily medications (p < 0.001). They were also more likely to have reported more illness symptoms, missed more hours of work, spent more days in bed, and had a longer illness duration (all p < 0.001). We did not identify differences by vaccination status at the time of infection, age, race/ethnicity, education, occupation, or smoking status.

Medical care was sought in 16% of reinfection SARS-CoV-2 infection (n = 106). Unadjusted analyses indicated medical care utilization increased as age increased (p = 0.01), was more likely in females (p < 0.001), healthcare personnel and other frontline/essential workers were more likely than first responders (p = 0.004) and increased as the number of medications increased (p = 0.01). Participants with higher numbers of symptoms (p < 0.001), more hours of work missed (p = 0.01), more days spent in bed (p < 0.001), and longer illness duration (p < 0.001) were also more likely to seek medical care during their reinfections. No differences were identified by vaccination status, race/ethnicity, education, number of chronic conditions, or smoking status.

In adjusted models for first SARS-CoV-2 infections, the only factors associated with healthcare utilization from any variant of infection were the number of illness symptoms, number of days spent at least half in bed, and illness duration (Table 2). Participants who tested positive for SARS-CoV-2 during the Origin/Alpha variant predominance were 16% more likely to seek care for each illness symptom they had (OR 1.16, 95% CI 1.00–1.36). For every day they spent at least half of the day in bed, they were 13% more likely to seek care (OR 1.13, 95% CI 1.02–1.33), and for each additional day that their illness lasted, they were 1% more likely to seek care (OR 1.01, 95% CI 1.00–1.04).

**Table 2:** Factors associated with healthcare utilization in 1276 first SARS-CoV-2 infection during enrollment in AZ HEROES

Characteristic	Origin/Alpha first infection n = 210		Delta first infection n = 121		Omicron first infection n = 945	
	OR^a^	95% CI^b^	OR	95% CI	OR	95% CI
**Vaccination status at infection**	0.76	0.33–1.64	0.98	0.49–2.02	1.12	0.88–1.43
**Age**	1.00	0.96–1.05	1.02	0.95–1.1	1.02	0.99–1.04
**Gender**	1.26	0.53–3.00	0.15	0.03–0.67	0.88	0.57–1.36
**Race/Ethnicity**	1.00	0.99–1.00	1.00	0.99–1.01	1.00	0.99–1.01
**Education**	0.65	0.29–1.47	1.81	0.46–7.91	1.20	0.75–2.00
**Occupation category**	1.00	0.56–1.78	0.75	0.31–1.79	0.91	0.72–1.13
**# of chronic conditions**	1.79	0.91–3.64	3.76	0.92–17.57	1.07	0.79–1.44
**Body mass index**	1.00	0.93–1.07	1.25	0.94–1.51	1.03	0.98–1.07
**Smoking status (tobacco and vaping)**	0.76	0.24–2.2	1.44	0.89–12.79	1.20	0.75–1.90
**# of daily medications**	0.91	0.61–1.33	0.68	0.35–1.21	1.16	0.98–1.38
**# of illness symptoms**	1.16*	**1.00–1.36**	1.12*	**1.00–1.49**	1.09*	**1.01–1.16**
**# of hours of work missed**	2.64	0.70–13.32	2.17	0.59–16.35	1.36	0.83–2.25
**# of days spent at least half in bed**	1.13*	**1.02–1.33**	1.23*	**1.00–1.59**	1.12*	**1.03–1.22**
**Illness duration (# of days)**	1.01*	**1.00–1.04**	1.01*	**1.00–1.03**	1.01*	**1.00–1.02**

a. OR = odds ratio, calculated with multivariable logistic regression models with robust standard errors. Models adjusted for all listed characteristics

b. CI = Confidence Interval

* statistically significant

In first infections from the Delta variant, participants were 12% more likely to seek medical care for every illness symptom they experienced (OR 1.12, 95% CI 1.00–1.49). For every day they spent at least half in bed, they were 23% more likely to seek medical care (OR 1.23, 95% CI 1.00–1.59), and for each additional day that their illness lasted, they were 1% more likely to seek care (OR 1.01, 95% CI 1.00–1.03).

If the first infection was from the Omicron variant, participants were 9% more likely to seek medical care for each additional illness symptom (OR 1.09, 95% CI 1.01–1.16), 12% more likely to seek care for each additional day they spent at least half in bed (OR 1.12, 95% CI 1.03–1.22), and 1% more likely to seek care for each additional day that their illness lasted (OR 1.01, 95% CI 1.00–1.02).

There were additional characteristics associated with higher medical utilization during Omicron variant reinfections compared to first infections from Origin/Alpha or Delta variants (Table 3). Participants were 4% more likely to seek medical care for each year older they were (OR = 1.04, 95% CI 1.01–1.07). Unlike first positives, there was no difference by number of illness symptoms, but participants were 124% more likely to seek care for each hour of work missed (OR = 2.24, 95% CI 1.11–4.74), 12% more likely for each day spent in bed (OR = 1.12, 95% CI 1.01–1.27), and 1% more likely for each additional day that the illness lasted (OR = 1.01, 95% CI 1.00–1.02). Adjusted models for Origin/Alpha and Delta reinfections were not able to be calculated due to limited observations.

**Table 3:** Factors associated with healthcare utilization in 599 Omicron SARS-CoV-2 reinfection during enrollment in AZ HEROESa

Characteristic	Omicron reinfection n = 599	
	OR^b^	95% CI^c^
**Vaccination status at infection**	0.99	0.76–1.31
**Age**	1.04*	**1.01–1.07**
**Gender**	0.45	0.21–0.91
**Race/Ethnicity**	1.00	0.98–1.01
**Education**	1.78	0.91–3.8
**Occupation category**	1.44	0.87–2.02
**# of chronic conditions**	0.77	0.45–1.3
**Body mass index**	1.03	0.98–1.09
**Smoking status (tobacco and vaping)**	0.63	0.27–1.39
**# of daily medications**	1.25	0.97–1.61
**# of illness symptoms**	1.06	0.96–1.18
**# of hours of work missed**	2.24*	**1.11–4.74**
**# of days spent at least half in bed**	1.12*	**1.01–1.27**
**Illness duration (# of days)**	1.01*	**1.00–1.02**

a. Odds ratios for the 26 Origin/Alpha reinfections and 22 Delta reinfections unable to be calculated due to limited observations and model convergence issues

b. OR = Odds Ratio, calculated with multivariable logistic regression models with robust standard errors. Models adjusted for all listed characteristics

c. CI = Confidence Interval

* statistically significant

## Discussion

This study examined healthcare utilization during first SARS-CoV-2 infection and reinfections in a large, diverse cohort of frontline and essential workers in Arizona from July 2020 through February 2023. The prospective nature of the study allowed for a unique comparison of healthcare utilization throughout the course of the pandemic in workers across an entire state, and to examine drivers of utilization for both first positives as well as reinfections. While overall healthcare utilization, and the subsequent strain on healthcare resources, has been greater during the COVID-19 pandemic than other recent pandemics, [17] the overall healthcare utilization rate amongst this cohort was relatively small. This is likely due to the overall healthy worker make-up of study participants and the high rates of vaccination in the cohort.

Influenced by conflicting previous literature that found SARS-CoV-2 reinfection contributed additional risk of death, hospitalization, and sequelae, [18] or not, [19, 20] the current analysis was stratified according to whether the SARS-CoV-2 infection was the participant’s first infection or a repeat infection. During first SARS-CoV-2 infection, we found participants infected with the Omicron variant had the lowest rate of healthcare utilization of all variants, which has not been the case with the general population [3, 4]. This was not the case in our cohort during reinfections, where individuals were most likely to seek care from Omicron reinfections. While this is in line with trends found in the wider public, this should be interpreted with caution as the number of reinfections from the Origin/Alpha or Delta variants in our cohort is limited. First infections and reinfections occurring with younger individuals, men, those with fewer chronic conditions, lower BMI, shorter illness, as well as fewer daily medications, illness symptoms, number of hours work missed, and number of days spent in bed all had lower rates of healthcare utilization. As nearly all of these are markers of better health or less severe illness, it would be expected that they would be associated with lower rates of healthcare utilization. In contrast to some previous findings, [18] the current analysis found lower rates of healthcare utilization for reinfections compared to first infections. It is unclear whether this might be due to differences in underlying participant characteristics – particularly age, gender, and race/ethnicity – or smaller geographic area of the current study population, or some other reason.

In adjusted models for first infections, we found no difference in healthcare utilization by vaccination status, sociodemographic characteristics, or participant baseline health characteristics by any variant of infection. The main drivers of healthcare utilization found in our analysis of first infections were related to the severity and duration of illness. Increasing number of symptoms, number of days spent at least half in bed, and duration of illness were associated with higher medical utilization in all variants of first infection.

In adjusted models for reinfection from the Omicron variant, increased age, hours of work missed, days spent in bed, and illness duration were all positively associated with healthcare utilization. Despite the relatively modest effect sizes of the ORs, their cumulative impact on healthcare can be contextualized by the incremental risk posed from single symptoms, number of hours work missed, number of days spent in bed, number of days of illness, and year of age. Given the wide distribution of these variables within the cohort, they represent a meaningful difference. While the literature examining overall healthcare utilization rates during the COVID-19 pandemic has been well established, [21–24] specific examinations on factors related to utilization in the wake of SARS-CoV-2 infection is not nearly as robust.

This study is subject to several limitations. First, because participants are all from the state of Arizona, there may be limited generalizability, though there was wide distribution by several sociodemographic and health characteristics. Second, the study population was of overall good health, and individuals willing to complete weekly nasal swabs and participate in a long prospective cohort may not be generalizable to the overall population. Third, all healthcare utilization was self-reported. It is possible that there were some individuals who did not report care that they received and may have been misclassified, though each participant had at least two opportunities to report any medical care they may have received. Fourth, the final illness survey was administered upon the cessation of symptoms. It is possible that some participants may have experienced a relapse in health and may have received medical care following their final illness survey.

## Conclusions

The AZ HEROES cohort allowed for a unique examination of the drivers of healthcare utilization throughout the different variants of the COVID-19 pandemic. Our findings suggest that efforts to ease the strain on healthcare systems might best be focused on decreasing symptom number and severity, and that efforts focused on increasing messaging to specific populations defined by sociodemographic characteristics might be less impactful. Continued efforts to encourage vaccination in order to decrease symptom severity [25–27] would seem helpful in decreasing, as would continued research into the development of antiviral treatments that show promise in reducing healthcare utilization when administered to SARS-CoV-2-positive patients at risk of severe disease but are experiencing mild to moderate symptoms at the time of administration [28].

## Data Availability

The datasets generated and/or analysed during the current study are not publicly available due to restriction by the Centers for Disease Control and Prevention but are available from the corresponding author on reasonable request.
